# Providing quality data in health care - almost perfect inter-rater agreement in the Norwegian tonsil surgery register

**DOI:** 10.1186/s12874-018-0651-2

**Published:** 2019-01-07

**Authors:** Siri Wennberg, Lasse A. Karlsen, Joacim Stalfors, Mette Bratt, Vegard Bugten

**Affiliations:** 1 Department of Medical Quality Registries, St. Olav’s University Hospital, MTFS, Torgarden, P.O. Box 3250, 7006 Trondheim, Norway; 2 Sheikh Khalifa Medical City, Ajman, United Arab Emirates; 3 Institute of Clinical Sciences, Sahlgrenska Academy at the University of Gothenburg, P.O. Box 426, 405 30 Göteborg, Sweden; 4 Department of Otorhinolaryngology, Head and Neck Surgery, St. Olav’s University Hospital, Sluppen, P.O. Box 3250, 7006 Trondheim, Norway; 5 Department of Neuromedicine and Movement Science, Norwegian University of Science and Technology, 7006 Trondheim, Norway

**Keywords:** Data quality, Medical quality register, Tonsil surgery, Chronic tonsillitis, Tonsillectomy, Tonsillotomy

## Abstract

**Background:**

The Norwegian Tonsil Surgery Register (NTSR) was launched in January 2017. The purpose of the register is to present data on tonsil surgery to facilitate improvements in patient care. Data used for evaluating the quality of medical care needs to be of high reliability. This study aims to assess the inter-rater reliability (IRR) of the variables reported to the register by medical professionals.

**Methods:**

The study population consists of the first 137 tonsil surgery patients who were included in the NTSR at St. Olav’s University Hospital in Trondheim. An experienced rater completed the register’s paper form for all 137 patients based on their electronic medical records, blinded for the data already in the register. To assess the inter-rater reliability between the register and the external rater, we calculated observed agreement, Cohen’s kappa and Gwet’s AC_1_ coefficients with 95% confidence intervals.

**Results:**

All tested variables in the NTSR have almost perfect reliability except for the variable for the *cold steel* technique, which had a substantial to almost perfect reliability. The inter-rater agreement was substantial to almost perfect for every variable, with substantial (kappa/AC_1_ > 0.61) to almost perfect (kappa/AC_1_ > 0.81) agreement for all the examined variables.

**Conclusion:**

This study shows that the reliability of the NTSR is high for all variables registered by the professionals at the hospital immediately after surgery.

## Background

There is an increasing demand from patients, health care providers and payers for transparency in healthcare [1]. Medical quality registers can be an important tool for quality improvement in health care, as well as a source of data for disease monitoring and clinical or epidemiological research. A register can measure results and compare results over time and between participating users. It can also be used to measure the results of specific quality improvement projects [2]. National quality registers can be said to be unique tools for follow-up and results assessment [3]. Data from medical quality registers with relevant and reliable results are used more and more in research and as a basis for forming public health policy [1]. Measuring quality is a crucial part of the shift towards value-based health care. By measuring the outcome of patient care, while at the same time recording the procedures and methods that are utilized, doctors, hospitals and medical communities as a whole have a tool for learning from each other. With this particular register data, results and research based on the data from the register is of interest to anyone who performs tonsil surgery, not only in Norway but also in the entire world [4].

To meet the demand from patients, health care providers and payers, the Norwegian Association for Otorhinolaryngology Head and Neck Surgery (NOLF) initiated the development of several Norwegian quality registers within the Ear, Nose and Throat (ENT) specialty in 2014. NOLF initiated the quality registers to improve ENT care and to facilitate patient-oriented ENT research. Additionally, the register can be used to monitor clinical practices in Norway as well as monitor the implementation of new techniques in the treatment of patients with tonsil diseases [5]. A quality register for tonsil surgery was the first national ENT quality register to be established. Across specialties, tonsil surgery is one of the most frequently performed operations in Norway, with considerable differences in clinical practices and outcomes throughout the country [6]. Approximately 10.000 tonsil surgery procedures are performed every year in Norway [7].

In September 2016, the Ministry of Health and Care Services in Norway accredited the Norwegian Tonsil Surgery Register as a national register, and in January 2017, the register became operational at St. Olav’s University Hospital in Trondheim. All Norwegian ENT-clinics, both public hospital units and private units, were encouraged to include patients and submit data. Inclusion started as a trial at St. Olav’s University Hospital in Trondheim, and throughout 2017 an increasing number of units started to submit data. As of February 2018, all public hospitals in Norway report data to the register [5].

The structure and variables of the NTSR are based on the National Tonsil Surgery Register in Sweden. The Swedish register was established in 1997 and includes patients from both public and private practitioners including more than 80% of all patients undergoing tonsil surgery since 2013 [8–11].

Data used to evaluate the quality of surgical care needs to be of high reliability to ensure valid quality assessment. It is crucial that the data is as correct as possible to be able to draw correct conclusions from a quality register [12]. Validation against source data such as medical records makes it possible to identify potential issues in one or more variables [13, 14]. Inter-rater reliability is the level of agreement between two or more individuals who measure or categorize the same objects or actions. The individuals who perform the measuring or categorization in an inter-rater reliability study are referred to as *raters.* Utilizing a nominal or ordinal scale the raters will categorize a set of objects or actions, and the degree to which the different raters put the same objects or actions in the same category is referred to as inter-rater reliability [15]. If the results show that a variable is systematically misinterpreted, the instructions and definitions of the variable may be clarified to resolve the issue. This is the first inter-rater reliability (IRR) study of the variables in the NTSR, and to our knowledge, there are no international publications on the inter-rater reliability of the variables from the Swedish register.

The NTSR contains variables reported by the surgeons and by the patients or their caregivers [5]. The aim of this study was to assess the reliability of the variables reported by the surgeons to the NTSR by studying the inter-rater reliability in a sample of 137 patients treated at St. Olav’s University Hospital in Trondheim.

## Methods

### The Norwegian tonsil surgery register

The register includes data from patients who undergo tonsillectomy or tonsillotomy with or without simultaneous adenoidectomy. The register collects data on the individual level from professionals and the patients or their caregivers. The data collected are age, gender, indication for surgery, date of surgery, type of care and surgery, technique used for surgery and haemostasis as well as patient reported outcome measures including postoperative haemorrhage. The patient reported outcomes recorded are composed of complications and relief of symptoms after surgery, and they are reported directly from the patients or their caregivers. See Table 1 for a list of the variables included in this study and their definitions [5].

**Table 1:** Variables registered in the NTSR with definitions

Variable	Definition
Date of birth Date of surgery	
Indication of surgery	
Airway obstruction/snoring/hypertrophic tonsils	Tonsils cause breathing disorder during sleep (parent reported)
Recurrent tonsillitis	At least three episodes of acute tonsillitis during last 12 months
Peritonsillar abscess	Peritonsillar abscess or peritonsillitis warranting emergency operation, or history of peritonsillar abscesses/peritonsillitis
Chronic tonsillitis	Prolonged inflammation of the tonsils (at least 3 months) affecting daily activities
Other	Free field to register other indications
Surgical Unit	
Day case surgery	No admission overnight
Overnight surgery	Prearranged overnight admission
Type of surgery	
Primary surgery	No previous tonsil surgery performed
Revision surgery	Tonsillectomy or tonsillotomy performed previously
Extent of surgery	
Tonsillectomy only	Extracapsular removal of tonsils
Tonsillectomy and adenoidectomy	Extracapsular removal of tonsils and removal of adenoid
Tonsillotomy only	Partial removal of tonsils
Tonsillotomy and adenoidectomy	Partial removal of tonsils and removal of adenoid
Surgical technique	
Cold steel	Procedure performed with cold instruments only, for example knife, scissors or elevator
Radiofrequency	Radiofrequency energy is used for cutting and coagulation
Diathermy scissors	Procedure performed with bipolar diathermy scissors, which can simultaneously cut and coagulate
Ultracision	Procedure performed with instrument, which simultaneously cuts and coagulates using ultrasonic vibration
Dissection with bipolar diathermy	Tonsils are dissected using bipolar diathermy
Other	Free field to register other techniques
Technique for haemostasis	
Infiltration with local anaesthetic and adrenalin	Haemostasis achieved with adrenaline vasopressor effect
Monopolar diathermy	Heat coagulation of the vessels using monopolar diathermy
Bipolar diathermy	Heat coagulation of the vessels using bipolar diathermy
Ligature	Suture used to stop haemorrhage
Suture ligature	Suture with needle used to stop haemorrhage
Radiofrequency	Haemostasis achieved using radiofrequency instruments
None	Haemostasis achieved with compression only
Other	Free field to register other techniques
Primary haemorrhage requiring intervention (Yes/No)	Any haemorrhage requiring intervention and occurring after extubation during initial hospital stay

Participants are included in the NTSR after signing a written informed consent form. Register data from the surgery are recorded through a standardized questionnaire typically filed electronically by the surgeon postoperatively. However, in some cases the surgeons fill in paper forms, and a dedicated secretary or nurse subsequently enters the data using a web-based form. A user manual provides definitions of the variables and data entries [16].

### Data collection

For the present study, we included the first 137 consecutive tonsil surgery patients who were registered in the NTSR at St. Olav’s University Hospital in Trondheim. The included patients underwent surgery between the 2nd of January and the 30th of June 2017. The study includes 137 of 144 patients who were treated at St. Olav’s University Hospital in Trondheim during this period. The coverage of the NTSR at St. Olav’s University Hospital for this period was 95%.

Several different raters report to the register. There are 24 surgeons employed at the ENT department, and 17 of them performed tonsil surgery during the period covered by this study. All 17 surgeons included patients in the register. No patients or surgeons were excluded from data collection. The surgeons either reported to the register themselves electronically or filled in a paper form that was later entered electronically by a dedicated nurse or secretary. In this study, everyone who reports to the register from St. Olav’s University Hospital in Trondheim is treated as one rater, as the data in the register are compared to the data collected by the external rater. The raters reporting to the register were not aware that their reporting was going to be tested at the time of their reporting.

To investigate the inter-rater reliability of the NTSR, the external rater collected the same information that was reported to the register on the same 137 patients based on their Electronic Medical Records (EMR) blinded for the data already in the register. Date of birth and date of surgery were excluded from the reliability test. Data from the EMR were recorded on individual paper forms and later entered into an electronic database (Microsoft Excel). The registrations were compared with the original registrations in the NTSR performed by the doctors/nurses/secretaries at the hospital. The external rater has a good knowledge of the register and its variables. When there was doubt about the content in the EMR, the external rater consulted an experienced physician at the ENT department that knows the register well but who has not filled in any of the original registrations herself. Three cases (3/137) were discussed until a consensus opinion on each case was determined. The data collection by the external rater for the study was conducted between September and October 2017.

### Statistical analysis

Cases in the study were identified without randomization from the database. The sample size was determined on the decision to include all the patients included in the register at St. Olav’s University Hospital in Trondheim during the period from January 2017 through June 2017. The Goodness-Of-Fit (GOF) procedure by Donner and Eliasziw states that when testing for statistical differences between moderate (0.40) and almost perfect (0.90) kappa values, sample size estimates ranging from 13 to 66 are required [17]. Our sample of 137 patients exceeds the requisite numbers to detect generalizable estimates of inter-rater reliability. The confidence intervals (CIs) of the results also confirm that the sample size is appropriate to detect estimates of inter-rater reliability [18].

All variables in the study are nominal variables*.* The inter-rater agreement is presented in terms of observed agreement, Cohen’s kappa and Gwet’s AC_1_ coefficients with 95% confidence intervals [15, 18, 19].

In situations where a large proportion of the ratings fall into the same category and very few ratings fall into other categories, a variable will have what is referred to as a skewed trait prevalence. A skewed trait prevalence in a variable will influence the kappa statistic and will lead to an artificially reduced kappa coefficient because it is designed to adjust for random agreement. The reduction in the kappa statistic is proportionally influenced by the degree of skewness in the trait prevalence [20, 21]. In the cases included in this study with discrepancies between the kappa and AC_1_ coefficients, the reliability was considered based on the AC_1_ coefficient and the observed agreement when a substantially skewed trait prevalence was observed. The AC_1_ coefficient is not affected by unbalanced trait prevalence [15, 18]. Distribution of trait prevalence for each variable is shown in Table 2.

**Table 2:** Trait distribution for each variable in the register (*n* = 137)

	Yes (medical records)	Yes (register)	No (medical records)	No (register)
Indication of surgery				
Airway obstruction/snoring/hypertrophic tonsils	74	73	63	64
Recurrent tonsillitis	39	33	98	104
Peritonsillar abscess	4	4	133	133
Chronic tonsillitis	19	23	118	114
Other	1	1	136	136
Surgical Unit				
Day case surgery	86	91	51	46
Overnight surgery	51	46	86	91
Primary surgery or revision surgery				
Primary surgery	134	134	3	3
Revision surgery	3	3	134	134
Extent of surgery				
Tonsillectomy only	57	56	80	81
Tonsillectomy and adenoidectomy	27	27	110	110
Tonsillotomy only	9	13	128	124
Tonsillotomy and adenoidectomy	44	41	93	96
Surgical technique				
Cold steel	29	38	108	99
Radiofrequency	0	0	0	0
Diathermy scissors	107	105	30	32
Ultracision	0	3	137	134
Laser	0	0	0	0
Dissection with bipolar diathermy	2	1	135	136
Other technique	0	0	0	0
Technique for haemostasis				
Haemostasis achieved with compression only	12	10	125	127
Infiltration with local anaesthetic and adrenalin	5	6	132	131
Monopolar diathermy	0	2	137	135
Bipolar diathermy	124	124	13	13
Ligature	0	0	0	0
Suture ligature	1	1	136	136
Primary haemorrhage requiring intervention (Yes/No)	1	1	136	136

IRR can be measured as a score between 0 and 1. High agreement between the raters equals high reliability in the data collection. With complete agreement, the IRR is 1 (or 100%), and with complete disagreement the IRR is 0 (0%). Several methods for calculating IRR exist, ranging from simple (e.g., percent agreement) to more complex (e.g., Cohen’s Kappa adjusting for random agreement and Gwet’s AC_1_ adjusting for random disagreement) approaches [15].

Kappa and AC_1_ coefficients with values ≤0.20 are interpreted as slight agreement, 0.21–0.40 as fair agreement, 0.41–0.60 as moderate agreement, 0.61–0.80 as substantial agreement, and values above 0.80 as almost perfect agreement [22–24].

The AgreeStat 2015.6 software was used for calculating the observed agreement, kappa and AC_1_ statistics.

## Results

We assessed the inter-rater reliability of the 18 variables in the NTSR recorded by the ENT surgeons at the hospital. The sample of 137 patients was 43.8% female (*n* = 60) and 56.2% male (*n* = 77). The age distribution was from 1 to 57 years, with a mean age of 10.7 years.

### Inter-rater reliability of the variables concerning surgical information

The agreement was deemed almost perfect for all variables concerning surgical information (Table 3). For *indication of surgery* the kappa of 0.87 and the AC_1_ of 0.91 indicated an almost perfect agreement. The variable *surgical unit* had a kappa of 0.96 and an AC_1_ of 0.93 indicating an almost perfect agreement.

**Table 3:** Inter-rater reliability for surgical information in the Norwegian Tonsil Surgery Register

	n	Obs.agr.	Kappa (95% CI)	AC_1_ (95% CI)
Indication of surgery	137	0.92	0.87 (0.80 to 0.94)	0.91 (0.85 to 0.96)
Surgical Unit	137	0.96	0.92 (0.85 to 0.99)	0.93 (0.87 to 0.99)
Primary or revision surgery	137	0.99	0.66 (0.21 to 1)	0.98 (0.96 to 1)
Extent of surgery	137	0.93	0.89 (0.83 to 0.96)	0.91 (0.85 to 0.96)

The *variable primary or revision surgery* had a kappa of 0.66. However, with an observed agreement of 0.99, an AC_1_ of 0.98 and a skewed trait distribution, it is clear that the kappa coefficient was artificially low. Thus, the agreement was considered almost perfect for this variable. The agreement was almost perfect for the *extent of surgery* variable with a kappa of 0.89 and an AC_1_ of 0.91.

### Inter-rater reliability of the variables concerning surgical technique

The agreement was deemed substantial to almost perfect for all variables concerning surgical technique (Table 4). Out of the seven categories for surgical technique, only four were used. Neither rater answered that *radiofrequency*, *laser* or *other techniques* were used. Several of the variables had an artificially low kappa coefficient due to skewed trait distribution.

**Table 4:** Inter-rater reliability for surgical technique in the Norwegian Tonsil Surgery Register

	n	Obs.agr.	Kappa (95% CI)	AC_1_ (95% CI)
Cold steel	137	0.92	0.78 (0.66 to 0.91)	0.87 (0.80 to 0.95)
Radiofrequency	137	–	–	–
Diathermy scissors	137	0.94	0.83 (0.72 to 0.95)	0.91 (0.85 to 0.97)
Ultracision	137	0.98	0.00 (0 to 0)	0.98 (0.95 to 1)
Laser	137	–	–	–
Dissection with bipolar diathermy	137	0.99	0.66 (0.04 to 1)	0.99 (0.98 to 1)
Other technique	137	–	–	–

The variable for *cold steel* had a kappa of 0.78 and an AC_1_ of 0.87, indicating a substantial to almost perfect agreement. *Diathermy scissors* had a kappa of 0.94 and an AC_1_ of 0.91, indicating almost perfect agreement. Due to an extremely skewed trait distribution, the variable *ultracision* had a kappa of 0.00. However, the AC_1_ was 0.98, and the observed agreement was 0.98, indicating an almost perfect agreement. The variable *dissection with bipolar diathermy* also had an artificially low kappa of 0.66 due to a skewed trait distribution. However, an AC_1_ of 0.99 and an observed agreement of 0.99 indicated almost perfect agreement.

### Inter-rater reliability of variables concerning technique for perioperative haemostasis

The agreement was deemed almost perfect for all variables concerning perioperative haemostasis (Table 5). Neither rater answered that *ligature* had been used. Several of the variables suffered from skewed trait distribution.

**Table 5:** Inter-rater reliability for technique for perioperative haemostasis in the Norwegian Tonsil Surgery Register

	n	Obs.agr.	Kappa (95% CI)	AC_1_ (95% CI)
Haemostasis achieved with compression only	137	0.97	0.80 (0.61 to 0.99)	0.97 (0.93 to 1)
Infiltration with adrenalin	137	0.99	0.91 (0.72 to 1)	0.99 (0.98 to 1)
Monopolar diathermy	137	0.99	0.0 (0 to 0)	0.99 (0.96 to 1)
Bipolar diathermy	137	0.96	0.75 (0.55 to 0.94)	0.95 (0.90 to 0.99)
Ligature	137	–	–	–
Suture ligature	137	1.00	1.00 (1 to 1)	1.00 (1 to 1)
Postoperative haemorrhage requiring intervention	137	0.99	0.00 (−0.01 to 0.00)	0.99 (0.97 to 1)

The variable *haemostasis achieved with compression* had a kappa of 0.80, an AC_1_ of 0.97 and an observed agreement of 0.97, indicating almost perfect agreement. *Infiltration with adrenalin* had a kappa of 0.91 and an AC_1_ of 0.99, indicating almost perfect agreement. The variable *monopolar diathermy* had an extremely skewed trait distribution, causing an artificially low kappa of 0.00. However, it had an AC_1_ of 0.99 and an observed agreement of 0.99, indicating almost perfect agreement. For *bipolar diathermy* the kappa was 0.75, the AC_1_ was 0.95 and the observed agreement was 0.96. Controlling for skewed trait distribution the coefficients indicate an almost perfect agreement. The variable *suture ligature* had a kappa of 1.0, an AC_1_ of 1.0 and an observed agreement of 1.0, indicating almost perfect agreement.

*Postoperative haemorrhage* had a kappa of 0.00, which was artificially low due to an extremely skewed trait distribution. An AC_1_ of 0.99 and an observed agreement of 0.99 indicated almost perfect agreement.

## Discussion

The variables included in the NTSR had substantial to almost perfect reliability. The inter-rater agreement was almost perfect for every variable except for the *cold steel* technique, which had a substantial to almost prefect agreement. This high documented reliability facilitates the use of the register to improve clinical practice and to use the data for research.

The variable for *indication of surgery* had a kappa of 0.87 and an AC1 of 0.91, indicating almost perfect agreement. The categories *recurrent tonsillitis* and *chronic tonsillitis* comprised most of the discrepancies in this variable (Table 2). For recurrent tonsillitis, the reason for this discrepancy may be that there is no defined ICD-10 code for recurrent tonsillitis, thus demanding interpretation from the rater. A similar reason may be valid for *chronic tonsillitis* as there is no international agreement about the definition, and the definition used in the NTSR may be vague, contributing to the discrepancies. These findings address the need for engaging the professional community in the process of creating common definitions.

The patients included in this study were younger than the average population that undergoes tonsil surgery in Norway. The mean age for the patients in our study was 10.7 years, while the mean age of all patients in the NTSR for 2017 was 15.3 years [25]. The mean age of all registered patients from 2013 to 2015 in the National Tonsil Surgery Register in Sweden was 13.3 years [8]. In some parts of Norway, young children are more often treated at public hospitals than in private practices, as is the case at St. Olav’s University Hospital in Trondheim. This explains why the patients in our study are younger than the population as a whole. As a result of these differences in indication for surgery and treatment between age groups, it is reasonable to assume that a sample with a significantly higher mean age would have more cases of disagreement on the variable for *indication for surgery,* specifically for the categories of *recurrent tonsillitis* and *chronic tonsillitis.* Both in Norway and internationally, younger children are more often treated for airway obstructions, while teenagers and adults more frequently undergo surgery because of infections.

The variable for the surgical technique *cold steel* had a kappa of 0.78 and an AC_1_ of 0.87, which indicates substantial to almost perfect agreement. The discrepancy between the external rater and the professional consists of the professional reporting to the register that *cold steel* was used, but the external rater did not find this in the EMR. This may be due to two or more techniques being utilized during the surgery, while it was not recorded as such in the EMR despite being reported to the register.

### Strengths and limitations

The complete recording of all 137 patients in the study group, with no missing values contributes to the strength of this study. The reason for this is that all variables are obligatory in the online form; it is not possible to finish the form without answering each question. This is facilitated by including few variables in the register, and the fact that it takes only 1–2 min per patient to register the data.

The study was performed after the first 6 months of collecting data which included 137 patients. This is a relatively short period of time and performing the study at a later stage could enable the study a larger scope. However, testing the quality of the data in the register is a continual process which is important to start as soon as possible [26]. The GOF-procedure also confirms that our sample exceeds the required sample size [17].

The results showed substantial discrepancies between the kappa and AC_1_ coefficients for multiple variables. When the variable had a skewed trait distribution, the kappa was considered artificially low, and the reliability of the variable was considered on the basis of the AC_1_ and observed agreement. A skewed trait distribution explained the discrepancies between the kappa and AC_1_ in every instance, and a strong agreement between the raters could therefore be confirmed. However, it is important to note that a skewed trait distribution means that the tested agreement concerns one of the categories in a variable more than the other categories.

Cold technique is the most frequently used technique for performing tonsillectomies in Norway [27]. Cold technique usually leads to less postoperative bleeding and less postoperative pain [3]. Nevertheless, a substantial amount of procedures in Norway are done with the use of warm instruments such as diathermy scissors, bipolar diathermy or radiofrequency. The reason for this is probably that the use of warm instruments causes less bleeding during surgery and less time in the operating theatre. The use of radiofrequency, laser and other surgical techniques are not often used in Norway, and these variables were not used by any rater at St. Olav’s University Hospital in Trondheim. This is presumably because there was no tradition of using these techniques during tonsil surgery at the hospital [27]. As a result, this study cannot determine whether there is strong agreement for these variables.

There are several raters; surgeons, nurses and secretaries, reporting to the register. In this study, these raters are treated as one, and it is conceivable that this may affect the results. One rater may report differently than the other, and it can be difficult to distinguish individual mistakes. However, the aim of this study was to measure the reliability of the register in a clinical practice with several different individuals registering data. Thus, this study is testing the reliability of the results reported by different raters. The individuals reporting to the register have read the same guidelines for reporting to the register. The effects of having multiple raters instead of a single rater are also mitigated by the fact that the sample size is far larger than required by the Donner and Eliasziw GOF approach [17]. The fact that the results of the study indicate almost perfect agreement on all variables in the register shows that the study design is not compromised by this factor.

As mentioned before, this study is important for documenting the reliability of data registered in the NTSR. To fully review the validity of the register, there are a number of studies needed. Naturally, it is also important to test the reliability of the patient reported outcome variables in the register. Other dimensions of data validity that need to be tested are comparability, completeness and timeliness. This study only includes patients from St. Olav’s University Hospital in Trondheim. In future studies, it will be important to include other hospitals and private units to see if the inter-rater reliability is the same across time and geographic areas.

A final factor to consider is that it is difficult to determine whether the agreement, or discrepancy, between raters is due to the quality of the hospitals electronic medical records, due to the quality of the variables in the register, the system for reporting to the register or to the quality of the registration by the raters.

## Conclusion

This study shows that the reliability of the NTSR is high for all variables that are registered at the hospital immediately after surgery. The information reported in the patient’s electronic medical records is the same as the information reported to the register. We found some small discrepancies in the variables for *indication for surgery* and for the variable *surgical technique*. This may indicate that there is a need for international agreed upon definitions to facilitate standardization about when to use recurrent tonsillitis or chronic tonsillitis as indications for surgery. The reason for the discrepancies in the variable surgical technique is likely related to detailed information in the register as compared to the patient journal. The high reliability of the NTSR makes it possible to use the data in quality improvement measures, research and as a basis for forming public health policy.

## Abbreviations

Cis: Confidence intervals
EMR: Electronic medical records
ENT: Ear, Nose and Throat
GOF: The Goodness-Of-Fit
IRR: Inter-rater reliability
NOLF: Norwegian Association for Otorhinolaryngology Head and Neck Surgery
NTSR: The Norwegian Tonsil Surgery Register
